# A Case of Coronary Vasospasm after Repeat Rituximab Infusion

**DOI:** 10.1155/2015/523149

**Published:** 2015-03-18

**Authors:** Calvin Ke, Amit Khosla, Margot K. Davis, Cameron Hague, Mustafa Toma

**Affiliations:** ^1^Department of Medicine, St. Paul's Hospital, University of British Columbia, 548-1081 Burrard Street, Vancouver, BC, Canada V6Z 1Y6; ^2^Division of Cardiology, St. Paul's Hospital, University of British Columbia, 479-1081 Burrard Street, Vancouver, BC, Canada V6Z 1Y6; ^3^Department of Radiology, St. Paul's Hospital, University of British Columbia, 2nd Floor, 1081 Burrard Street, Vancouver, BC, Canada V6Z 1Y6

## Abstract

Coronary artery vasospasm (CAV) can be triggered by medication reactions. CAV occurring after multiple exposures to rituximab has not been previously described. A 61-year-old woman with no cardiac risk factors was treated with the sixth cycle of gemcitabine, cisplatin, dexamethasone, and rituximab therapy. Fifteen minutes after rituximab infusion commenced, she developed typical cardiac chest pain with ST segment elevations on electrocardiogram. Angiogram revealed evidence of coronary vasospasm. The patient was successfully treated with amlodipine. This case underlines the importance of monitoring cardiac side effects of rituximab therapy, even after multiple cycles.

## 1. Summary

A 61-year-old woman with no cardiac risk factors was treated with the sixth cycle of chemotherapy. Fifteen minutes after rituximab infusion commenced, she developed typical cardiac chest pain with ST segment elevations on electrocardiogram. Angiogram revealed evidence of coronary vasospasm. This case underlines the importance of monitoring cardiac side effects of rituximab therapy, even after multiple cycles.

## 2. Background

Although coronary artery vasospasm (CAV) is a known cause of myocardial ischemia, infarction, and malignant arrhythmia, the triggers for and mechanisms of CAV are not well defined. Vascular smooth muscle hyperreactivity, endothelial dysfunction, and altered autonomic function are proposed mechanisms of coronary artery spasm causing near or total occlusion [1]. Among documented causes of CAV, medication reactions are known to trigger attacks.

We report a case of CAV occurring during chemotherapy with rituximab and discuss its significance for other patients receiving rituximab.

## 3. Case Presentation

A 61-year-old woman with no cardiac risk factors nor cardiac history was admitted to hospital to receive her sixth and final cycle of gemcitabine, cisplatin, dexamethasone, and rituximab (GDP-R) for diffuse large B-cell lymphoma (DLBCL). She received dexamethasone 40 mg PO in the morning, followed by dexamethasone 8 mg IV prior to gemcitabine 1640 mg IV over 30 minutes with no adverse reaction. An hour later, she received rituximab IV at 100 mg/h, with routine diphenhydramine 30 minutes before.

**Figure 1:**
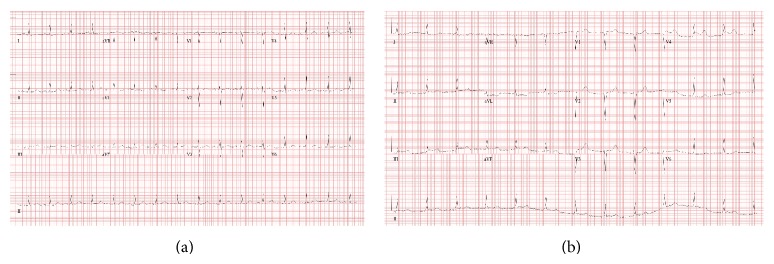
Electrocardiograms: (a) at baseline; (b) after chest pain onset.

**Figure 2:**
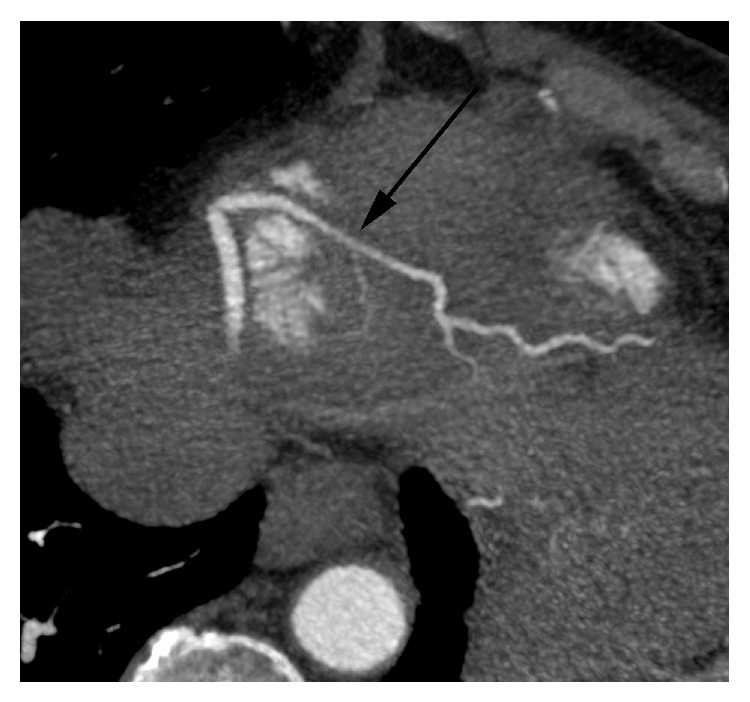
4.5 mm maximum intensity projection curved multiplanar reformatted cardiac CT image demonstrating smooth narrowing of proximal/mid portion of early arising PDA with no discernable plaque.

Fifteen minutes after starting the rituximab infusion, she developed moderate intensity (rated 5/10) retrosternal chest pain radiating to the left arm associated with diaphoresis and two vomiting episodes. Immediate electrocardiogram revealed ST segment elevations in the inferior leads with reciprocal lateral ST segment depressions (Figures 1 and 2). She was treated with 0.5 mg hydromorphone and rituximab was stopped. Her pain resolved in 5 minutes without further therapy, and the cardiology team was consulted.

After initiating aspirin and clopidogrel, she received an emergent angiogram to rule out thrombotic occlusion of a coronary vessel. Angiogram findings revealed spontaneous posterior descending artery (PDA) spasm, distal left anterior descending (LAD) artery tapering, and mild coronary artery disease (CAD) in mid-LAD artery, without evidence of thrombotic occlusion. The PDA vasospasm improved dramatically with intracoronary nitroglycerin (no record of ECG changes or symptoms during angiography). Ejection fraction was 60% and left ventricular end diastolic pressure was 6 mm Hg. Further chemotherapy was discontinued. Troponin I rose from 0.04 *μ*g/L two hours after the episode, peaking at 0.22 *μ*g/L within 24 hours of the episode. Other laboratory tests were unremarkable aside from LDH 579 units/L. She was treated with amlodipine and her chest pain did not recur during the admission.

Her past medical history was significant for chronic lymphocytic leukemia diagnosed in 2008 with transformation in 2012 to DLBCL. She had previously been treated with six cycles of cyclophosphamide, doxorubicin, vincristine, prednisone, and rituximab (CHOP-R) with methotrexate for CNS relapse, all without any known cardiotoxicity. She had no prior chest radiation. Previous GDP-R treatments were similarly uneventful, although, in retrospect, she vaguely recalled having chest discomfort during previous infusions which she did not report because the pain was mild and resolved spontaneously. Echocardiogram performed prior to chemotherapy initiation documented a structurally normal heart with normal ejection fraction.

Cardiac CT angiogram (CCTA) was performed to rule out extraluminal atherosclerotic disease. The results showed no obvious epicardial coronary disease. However, attenuation was noted in the PDA with abnormal soft tissue appearing to infiltrate the epicardial space, possibly representing lymphoma.

## 4. Discussion

This report represents the first case of CAV occurring in association with a temporal relationship to rituximab infusion after multiple prior uneventful exposures to rituximab. While CAV occurs most commonly due to 5-fluorouracil and capecitabine, adverse reactions to rituximab are extremely rare, with only one other case reported [2].

Rituximab is a monoclonal antibody to CD20 which is used to deplete B-cells. Infusion reactions to rituximab and other monoclonal antibodies occur via an unknown mechanism usually during the first infusion, but 10–30% of reactions can be delayed to subsequent infusions [3]. Our patient developed a reaction in her twelfth cycle of rituximab.

Although CAV patients are typically found to have no atherosclerotic disease on conventional angiography, it is well documented that segments with atherosclerotic plaque are prone to vasospasm due to endothelial dysfunction. Given the inability of angiography to visualize extraluminal atherosclerosis, we proceeded with CCTA.

In this case, we cannot completely exclude other causes of CAV including a combined effect of concomitant antineoplastic agents (i.e., gemcitabine) or lymphoma [4]. Further imaging with a PET scan was planned to assess the possibility of lymphomatous infiltration increasing susceptibility to PDA spasm. Unfortunately, the patient passed away from lymphoma before this could occur, and no autopsy was performed.

This case underlines the importance of close monitoring of patients undergoing chemotherapy with rituximab for adverse cardiac reactions including CAV and those ongoing vigilance after the first infusion is necessary for timely management.

## Abbreviations

CAD: Coronary artery disease
CAV: Coronary artery vasospasm
CCTA: Cardiac CT angiogram
CHOP-R: Cyclophosphamide, doxorubicin, vincristine, prednisone, and rituximab
DLBCL: Diffuse large B-cell lymphoma
GDP-R: Gemcitabine, cisplatin, dexamethasone, and rituximab
LAD: Left anterior descending artery
PDA: Posterior descending artery.
